# Delivery and use of individualised feedback in large class medical teaching

**DOI:** 10.1186/1472-6920-13-63

**Published:** 2013-05-03

**Authors:** Steven A Burr, Elizabeth Brodier, Simon Wilkinson

**Affiliations:** 1 Plymouth University Peninsula Schools of Medicine & Dentistry, Portland Square, Drake Circus, Plymouth, Devon PL4 8AA, UK; 2 School of Biomedical Sciences, Medical School, Queen’s Medical Centre, University of Nottingham, Nottingham NG7 2UH, UK; 3 Information Services Research and Learning Resources Division, King’s Meadow Campus, University of Nottingham, Nottingham NG7 2NR, UK

**Keywords:** Feedback, Objective, Assessment, Blueprint

## Abstract

**Background:**

Formative feedback that encourages self-directed learning in large class medical teaching is difficult to deliver. This study describes a new method, blueprinted feedback, and explores learner’s responses to assess its appropriate use within medical science teaching.

**Methods:**

Mapping summative assessment items to their relevant learning objectives creates a blueprint which can be used on completion of the assessment to automatically create a list of objectives ranked by the attainment of the individual student. Two surveys targeted medical students in years 1, 2 and 3. The behaviour-based survey was released online several times, with 215 and 22 responses from year 2, and 187, 180 and 21 responses from year 3. The attitude-based survey was interviewer-administered and released once, with 22 responses from year 2 and 3, and 20 responses from year 1.

**Results:**

88-96% of learners viewed the blueprinted feedback report, whilst 39% used the learning objectives to guide further learning. Females were significantly more likely to revisit learning objectives than males (p = 0.012). The most common reason for not continuing learning was a ‘hurdle mentality’ of focusing learning elsewhere once a module had been assessed.

**Conclusions:**

Blueprinted feedback contains the key characteristics required for effective feedback so that with further education and support concerning its use, it could become a highly useful tool for the individual and teacher.

## Background

Feedback acts as a response to performance; correcting and reinforcing knowledge to minimise error. When feedback is used within teaching it can bring learners through the stages depicted in the conscious competence theory [1], particularly from the important stage of unconscious incompetence to conscious incompetence. With further self-directed learning, this will hopefully lead to conscious competence and corrected understanding; the fundamental purpose of feedback. Without development to this stage of learning and correction, the learner struggles to progress.

The cycle of learning proposed by Kolb [2] begins with the learner experiencing; “what do I know?”, which leads on to reflecting on the task; “what do I need to know?”. The cycle continues by thinking and conceptualising; “how much and how well do I understand?”, which when practiced effectively highlights areas that are partially understood, causing the learner to then correct their knowledge in the acting stage; “how can I take my learning further?”. The challenge lies in how the teacher can lead students individually through this cycle.

The literature suggests a number of feedback models that guide the individual through the cycle described by Kolb [2]. Models implemented within clinical teaching include Pendleton’s rules [3], ALOBA [4], the Chicago model [5], SET-GO [6], the SCOPME model [7] and the six-step problem-solving model [8]. With frequent opportunities for teacher-facilitated formative feedback during clinical training, these are appropriate feedback models to be used according to the teacher’s preferences and abilities. Furthermore, research is currently evaluating the benefits of feedforward interventions in the clinical setting to improve learner’s performance, through the learner focusing on internal standards and previous performance that they considered good practice [9]. However, these teacher-facilitated feedback models place a high demand on resources [10]. As a result, lecture-based medical teaching requires the development of alternative feedback models that meet the need to offer a universal feedback report to a large number of learners, whilst incorporating the key characteristics of effective feedback. Research suggests that for feedback to be effective it should have the characteristics of being: timely [11,12], specific [13], non-evaluative [6,14], individualised [15] and constructive [14,16]. This study explores learner’s responses to blueprinted feedback. This is an innovative feedback model that follows summative assessment in medical science teaching, providing a tool which offers feedback to large cohorts. This model has been repeatedly used throughout our medical curriculum and was recently commended as an example of good practice in advice supplementary to *Tomorrow’s Doctors*[17], but has not been explained until now.

### Blueprinted feedback

**Figure 1:**
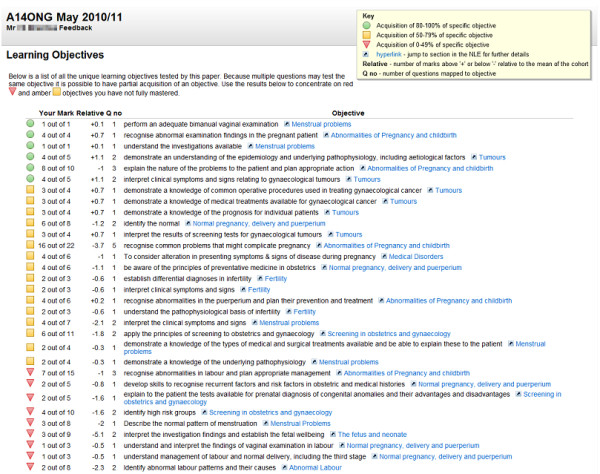
Example of a blueprinted feedback report received by an individual learner following a summative assessment.

Learning objectives establish what is expected of the learner and can be used to help monitor the learner’s progress in achieving what is expected. Mapping summative assessment items to their relevant learning objectives creates a blueprint. This blueprint can be used on completion of the assessment to automatically create a report which lists the module learning objectives examined in the assessment, ranked according to the individual’s achievement of each specific objective (Figure 1). The blueprinted feedback model offers an automated yet personalised option for delivering feedback to large numbers of learners. Presenting information as learning objectives has two key advantages: 1) it protects the validity of assessment items for future use, and 2) the format also has the potential to encourage deeper learning; drawing the learner’s attention away from the extraneous contextual detail of assessment questions, and directing their reference to the learning objectives. This focuses future learning and revision on learning objectives, leading to competence across the medical curriculum.

### Benefits for the learner

Historically, after a summative assessment often all that was fed back to students was a percentage pass mark. Blueprinted feedback offers a relevant report to learners at varying levels of competence. Through ranking the learning objectives according to achievement, it highlights strengths to the weak learner, and weaknesses to the strong learner. This form of constructive feedback affirms learners whilst stimulating self-correction.

### Benefits for the teacher

Blueprinting saves time. It is quicker to blueprint a summative assessment once than meet with students of a large cohort individually. Secondly, blueprinting adds additional perspective. As well as ticks/crosses on an assessment paper, linking this to learning objectives is very powerful when meeting a tutee and trying to generalise potential areas of weakness. Finally, a ‘by product’ of blueprinting to generate the personalised reports is that blueprinting allows mapping of the objectives *sampled* in the current assessment. This can be used over several sessions to ensure that all objectives are fully assessed.

**Figure 2:**
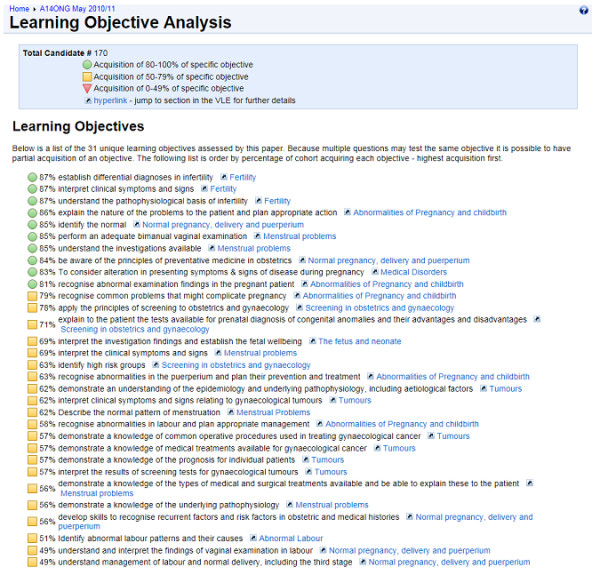
Example of a staff summary blueprinted feedback report for an entire cohort.

Highlighting the achievement of learning objectives in the feedback can also be used to offer a level of feedback to the teacher. A summary blueprinted report of the average performance for the whole cohort (Figure 2) gives the ability to generalise trends in the acquisition of objectives. As each assessment item is mapped to a learning objective and the teaching session it was taught in, it offers a clear picture to the teacher of what content was well communicated and understood by the learners. Whilst revealing areas of the curriculum that were thoroughly understood by the learners, this also highlights gaps in teaching, or the lack of resources available needed to support certain content. Further analysis of the cohort’s performance can reveal trends in weaker or stronger learners, providing insight into the cohort’s specific needs for future teaching.

### Aims

To describe a new computational procedure for automated delivery of individualised feedback to students following summative e-assessment. To evaluate the utility of the model by feedback from surveys of the student experience.

## Methods

### The feedback structure

The report (Figure 1) is specific, non-evaluative and individualised in its structure and format. Delivery can be via automated email or online network and triggered to be released immediately once the assessment is completed by the student, or delayed to be coincident with the release of assessment results as appropriate. With conflicting suggestions in the literature regarding the most effective time to offer feedback (i.e. immediately after the assessment, or later with the results), this model allows flexibility and the discretion of the faculty in the time of its release. The final characteristic of effective feedback is to be constructive. This is achieved by the electronic tags alongside each learning objective which link to the teaching session it was taught in, with additional supporting online resources available within the virtual learning environment. This guides the learner to correct or concrete knowledge, and complete the full cycle of learning.

We have also explored the efficacy of a report that uses a traffic light system to indicate the achievement of the learning objective. Fulfilled objectives are depicted by green traffic lights, partially fulfilled objectives light amber, and cause for concern light red. The e-assessment management software which facilitates this blueprinted feedback was developed in-house but is now available free as an Open Source Project at: http://rogo-oss.nottingham.ac.uk/.

All examinees within a cohort see the same objectives in their feedback report. However, the order of these objectives is ordered by personal performance on the questions linked to each objective – best at the top to worst at the bottom. The shape of the traffic light icons has also been changed to aid accessibility (i.e. colour blindness). The scale for determining which icon to display is shifted upwards as it is expected that most students will exceed 40-50%. Initially a straight 33%/66% split between red, amber and green was considered. However, it was thought inappropriate to award a green, and potentially psychologically very positive icon, for 67%. Instead the boundaries were shifted upwards to 50% and 80%.

We have also evaluated how well blueprinted feedback is received, understood and used. When analysing the extent that blueprinted feedback is utilised by learners currently studying undergraduate medicine, the following hypotheses were tested:

1. Learners highly value blueprinted feedback.

2. Learners use blueprinted feedback to assist in self-correction of knowledge.

### Data collection and analysis

Responses from medical students in years 1 and 2 of training were collected through an online, behaviour-based survey. As responders chose to self-complete the survey there was a larger response the first time it was released (samples of 180-215 from a population of 250), but a smaller response the subsequent time (sample size 21-22). The second survey explored the attitudes concerning use of blueprinted feedback, and targeted three year groups through an interviewer-administered survey (samples of 20-22). As a result, the views of three year groups were recorded to show differences in attitude at each stage of study (Table 1).

**Table 1:** Sample size from both surveys

**Survey**	**Year of entry (cohort)**	**Stage of study when surveyed**	**N**	**Gender**		**Total**
				**Male**	**Female**	
**1. Behaviour-based**	2008	Yr 1 S 1	187	68	119	187
		Yr 2 S 3	180	70	110	180
		Yr 2 S 4	21	7	14	21
	2009	Yr 1 S 1	215	71	144	215
		Yr 1 S 2	22	5	17	22
**2. Attitude-based**	2008	Yr 3 S 5	22	11	11	64
	2009	Yr 2 S 3	22	11	11	
	2010	Yr 1 S 1	20	10	10	

The total can only be summed in the attitude-based survey as responders from the behaviour-based survey overlap. The stage of study names are abbreviated from “Year x Semester y” to “Yr x S y”.

Questions were designed to avoid bias from order effect, acquiescence, central tendency and pattern answering [18,19]. The threshold for statistical significance was set at p < 0.05. Parametricity was determined using Shapiro-Wilks test, with appropriate further statistical tests used dependent upon the data distribution, variance and type.

## Results

### The value of blueprinted feedback within medical education

The responders across the three year groups averaged the value of feedback as 4.19 on a 5 point Likert scale (1 = not useful, 5 = very useful). The importance placed on receiving feedback following summative assessment was reflected by 88-96% of the cohort viewing the specific modular blueprinted feedback reports as they are released.

### Utilising blueprinted feedback to assist in self-correction

**Figure 3:**
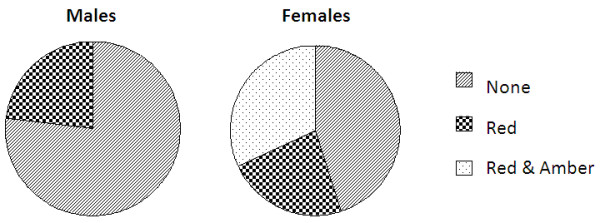
**Revisited traffic lights.** Pie charts showing which traffic light objectives learners in years 2 and 3 chose to revisit lecture content for. N = 44.

39% of the responders utilised the blueprinted feedback report to direct further learning and correction. There was a significant correlation (p = 0.012) between gender and level of use. 32% of the total female learners reflected upon both the red and amber light learning objectives, and revisited lecture content of both to improve knowledge. However, no males used the report to this depth, with 23% only revisiting the lecture content of learning objectives lit red. In summary, 77% males did not revisit any lecture content at all, compared with 45% of females who did not revisit any (Figure 3).

**Figure 4:**
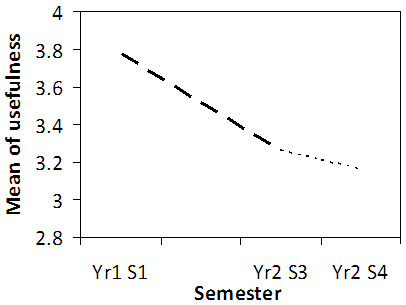
**Usefulness of feedback.** Line graph showing how highly responders from the 2008 entry cohort rated the usefulness of blueprinted feedback across successive semesters. No data collected in Yr1 S2. N = 187, 180, 21.

**Figure 5:**
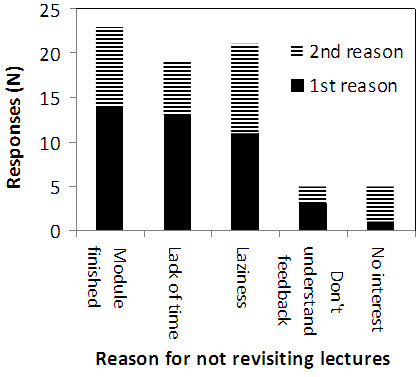
**Reasons for not revisiting unachieved objectives.** Stacked bar chart showing the primary and secondary reasons responders gave for not revisiting the learning objectives which they had performed poorly on. N = 44.

Overall, 61% of learners did not use the blueprinted feedback report for its intended purpose, and when considering the attitudes of students across the three years of study it is clear that their evaluation of the usefulness of the report declined with progression through the course (Figure 4). Possible barriers hindering the use of the blueprinted report were explored, with learners stating that the main factors influencing their use of the feedback are that the module had finished, a lack of time, and laziness (Figure 5).

The motivation of learners was also addressed in the survey, with results showing that the mean assessment grade students considered to reflect an adequate performance was 63.1%. 58% of responders then suggested they would be more motivated to improve and utilise the feedback report if they performed 10% lower than the year’s average (calculated as 65%), rather than if they achieved 55%.

## Discussion

### Use of blueprinted feedback

Blueprinted, objective-based feedback is unique in its ability to offer highly individualised, specific feedback following summative assessment that includes a depth of quantitative details without compromising assessment item validity. 88-96% of the responders viewed the feedback reports, evidencing a desire for individualised feedback, whilst in other studies only 46% of learners sought the individualised feedback [20].

This study showed only 39% of the learners utilised the blueprinted feedback report as extensively as intended, suggesting there are hidden obstacles to use. The unfamiliarity of the report causes some learners to instantly reject the feedback, highlighting the concrete need for education on the unique blueprinted format to reduce cognitive loading. Research shows that teaching medical students to learn how to give and receive feedback in the first year of their training is effective in instilling the self-reflective practice that is needed for a life-long career [21]. Feedback is a form of reflective practice; a skill that doctors must develop to benefit patients [22]. The study shows responders view blueprinted feedback as less useful over time, which is likely to be linked to the lack of education corresponding to the feedback report. Integrating teaching into the medical curriculum on feedback and on applying all stages of the learning cycle appears necessary.

Feedback from this group of learners is also being implemented, such as delaying the timing of the delivery to coincide with the assessment result. Additional levels of quantitative detail that reflect the cohort’s performance on specific objectives are being integrated into the report, after considering how learners appear to be motivated by norm-referencing. The responders demonstrated that the standard they hold for themselves is influenced more by comparison with peer performance than internal standards. This supports the use of feedback that involves an external standard and motivation, rather than feedforward, which focuses on an internal standards and consequent motivation [9].

### Challenges facing the efficacy of blueprinted feedback

Research suggests that the impact feedback has upon learning is complex, with meta-analysis showing that in one third of experiments feedback interventions reduce performance [23]. Our surveys highlighted a number of factors that inhibited continued learning and correction. The predominant reason learners gave, “the module has finished”, reveals a hurdle mentality in learners who prematurely close the cycle of learning, before reflecting upon their feedback from summative assessment. This supports a greater emphasis on spiral curricula together with early signposting to students of objectives that will be revisited and built upon.

As research has explored the complexity behind learner stimulation and self-directed action, conflicting theories have developed. White proposes undifferentiated grading systems that only indicate whether the learner has passed or failed, to effectively stimulate improvement by reducing comparison and peer-competition [24]. Conversely, Hewson demonstrates that specificity and facts are a key element of effective feedback [14]. Kluger proposes that stimulation to improve depends on the learner’s state of mind with relation to the activity; whether it was for pleasure or to avoid pain [9]. However this theory relies heavily on the learners individual mindset and focus, and so is difficult to incorporate in large-scale feedback as it requires similar amounts of personal interaction as other widely accepted feedback models (Pendleton’s rules [3], ALOBA [4], the Chicago model [5], SET-GO [6], the SCOPME model [7], and the six-step problem-solving model [8]).

The development of blueprinted feedback has provided a tool that can be utilised in education broader than medicine. Indeed, at the University of Nottingham blueprinted feedback has been used in Engineering since the 2010/11 session and is now being explored by other disciplines. It assists moving the educational research field forward by offering a model that provides automated universal feedback which is both individualised and specific following summative assessment. The main factor restricting the use of blueprinted feedback is the lack of integrated education in the curriculum regarding the application of feedback and the blueprinted format itself. However, the feedback report incorporates the key elements required to become highly useful to the learner, when appropriately used within large class teaching.

## Conclusions

Medical students seek detailed feedback, but most do not use the feedback they receive effectively. Mapping the assessment items to the learning objectives enables incorporation of both qualitative and quantitative feedback, without compromising summative assessment bank questions. Thus the blueprinted report helps learners track their mastery of the objectives and in turn monitor the effectiveness of their learning, encouraging reflection on the learner’s strengths and weaknesses, while also guiding them towards further online resources. It may be possible to encourage reflection by providing a formative e-assessment where the blueprinted feedback would undergo blinded peer-review with the purpose of getting peer recommendations on how to improve performance. This would provide the opportunity for collaborative interaction and motivate students to spend time analysing the reports prior to a summative end of term e-assessment which could then test the same objectives but using different questions. In addition, a summary report for the entire cohort can present generalised trends to the teacher, to improve the future teaching of objectives.

## Competing interests

The authors declare that they have no competing interests.

## Authors’ contributions

SAB conceived the study and drafted the manuscript. SAB and EB designed the student surveys and interpreted the findings. EB conducted the student surveys, analysed the data and helped draft the manuscript. SW conceived, produced and refined the computational method. All authors read and approved the final manuscript.

## Pre-publication history

The pre-publication history for this paper can be accessed here:

http://www.biomedcentral.com/1472-6920/13/63/prepub
